# Targeting tumor microenvironmental barriers to enhance immunogenic cell death in solid tumors

**DOI:** 10.3389/fimmu.2025.1672601

**Published:** 2025-09-11

**Authors:** Jifeng Zhang, Wei Han, Mingchen Zhang, Yingjie Yi, Mei Long

**Affiliations:** ^1^ Zibo Central Hospital, Zibo, China; ^2^ Department of Oncology, Zibo Central Hospital, Zibo, China; ^3^ Department of Hematology, Zibo Central Hospital, Zibo, China; ^4^ Department of Pediatrics, Zibo Central Hospital, Zibo, China; ^5^ Department of Internal Medicine, Zibo Central Hospital, Zibo, China

**Keywords:** immunogenic cell death (ICD), tumor microenvironment (TME), immune suppression, tumor microenvironmental barriers, metabolic reprogramming

## Abstract

Immunogenic cell death (ICD) effectively triggers adaptive immune responses against cancer, yet its clinical application in solid tumors is hindered by tumor microenvironment (TME) barriers. These include immunosuppressive cell populations, dense extracellular matrix, abnormal vasculature, hypoxia, and metabolic suppression, which collectively impede immune infiltration and function. This review evaluates current therapeutic strategies to overcome these barriers, including vascular normalization (restoring abnormal tumor blood vessels to a more structured and functional state to improve perfusion and immune cell infiltration), extracellular matrix (ECM) modulation, alleviation of hypoxia, metabolic reprogramming, immunosuppressive cell targeting, physical remodeling, and nanoparticle-based drug delivery. Clinical evidence highlights the potential of these integrated approaches to enhance ICD-induced antitumor immunity, suggesting promising avenues for improving patient outcomes through combined modulation of the TME and ICD induction.

## 1 Introduction

Immunogenic cell death (ICD) has emerged as a therapeutic strategy that initiates adaptive immune responses against cancer (1, 2). By promoting dendritic cell (DC) activation, antigen presentation, and cytotoxic T-cell priming, ICD effectively turns dying tumor cells into an *in situ* vaccine, harnessing the patient’s own immune system to combat malignancies (3–5). However, despite advances in understanding ICD mechanisms and applications, the clinical translation of ICD-based therapies, particularly in solid tumors, remains limited due to the presence of multiple TME barriers (6).

The TME of solid tumors is immunosuppressive, structurally dense, and metabolically hostile. It comprises immunosuppressive cell populations, including regulatory T cells (Tregs), tumor-associated macrophages (TAMs), and myeloid-derived suppressor cells (MDSCs), which collectively suppress effector immune cell infiltration and function (7, 8). Additionally, physical constraints, such as dense extracellular matrix deposition, fibrosis, and abnormal tumor vasculature, further impede immune cell trafficking and infiltration, thereby reducing the therapeutic potential of ICD (9, 10). Furthermore, metabolic factors, including hypoxia, nutrient scarcity, and accumulation of immunosuppressive metabolites (adenosine, lactate), impair T-cell survival and function within the TME (11, 12).

Therefore, a crucial challenge—and equally significant opportunity—lies in targeting and overcoming these microenvironmental barriers to realize the therapeutic potential of ICD in solid tumors (2, 13). In this review article, we discuss the existing tumor microenvironmental obstacles that limit ICD efficacy, highlight emerging therapeutic strategies to dismantle these barriers, and underscore the clinical promise of strategically combining microenvironment-targeted therapies with ICD induction to significantly enhance cancer immunotherapy outcomes.

## 2 Tumor microenvironmental barriers impairing ICD effectiveness

Despite the promising therapeutic potential of ICD in activating antitumor immunity, its clinical effectiveness in solid tumors is severely hampered by complex microenvironmental barriers (14). These barriers encompass immunological, physical, and metabolic factors that collectively restrict immune cell infiltration, activation, and sustained function within tumor sites.

Firstly, the immunosuppressive cellular environment impairs ICD-induced immune activation. Solid tumors are populated by immunosuppressive cell types such as Tregs, TAMs polarized toward the anti-inflammatory M2 phenotype, myeloid-derived suppressor cells (MDSCs), and cancer-associated fibroblasts (CAFs) (15). These cells produce various immunosuppressive mediators, including transforming growth factor-beta (TGF-β), interleukin-10 (IL - 10), and vascular endothelial growth factor (VEGF), which collectively inhibit dendritic cell (DC) maturation, reduce cytotoxic T-cell proliferation, and dampen effector immune responses. Consequently, even when ICD successfully generates immunogenic signals, their translation into meaningful clinical responses remains suboptimal due to these immunological checkpoints within the TME (16–18).

Secondly, physical barriers posed by the TME also present significant hurdles to effective immune cell trafficking and infiltration. An extensively developed extracellular matrix (ECM, the network of structural proteins such as collagen and glycosaminoglycans that provides physical support to tissues), characterized by dense collagen fiber networks and high levels of hyaluronan, physically obstructs immune cell penetration and reduces the diffusion of therapeutic agents into tumor cores (19–21). Tumor fibrosis, driven largely by activated CAFs, further exacerbates this issue by enhancing ECM rigidity and reducing the functional perfusion of intratumoral vessels (22). Aberrant and tortuous tumor vasculature, featuring dysfunctional endothelial cells and compromised lymphatic drainage, further limits immune cell entry and migration, thereby maintaining the immune-excluded or immune-desert phenotype characteristic of many solid tumors.

Thirdly, metabolic alterations within the TME restrict immune cell function and survival, thereby limiting the effectiveness of ICD-induced immune responses. Hypoxia, resulting from poor vascular perfusion and rapid tumor cell proliferation, triggers tumor adaptation through hypoxia-inducible factors (HIFs), leading to increased anaerobic glycolysis (Warburg effect), excessive lactate production, and acidification of the tumor milieu (23). The accumulation of metabolites such as lactate, adenosine, and kynurenine directly suppresses cytotoxic T-cell activation, proliferation, and effector functions, while promoting the differentiation and function of immunosuppressive cells (24). Nutrient depletion (glucose, amino acids such as tryptophan and arginine) further impairs the metabolic fitness and persistence of infiltrating immune cells, ultimately compromising antitumor immunity following ICD induction.

**Figure 1:**
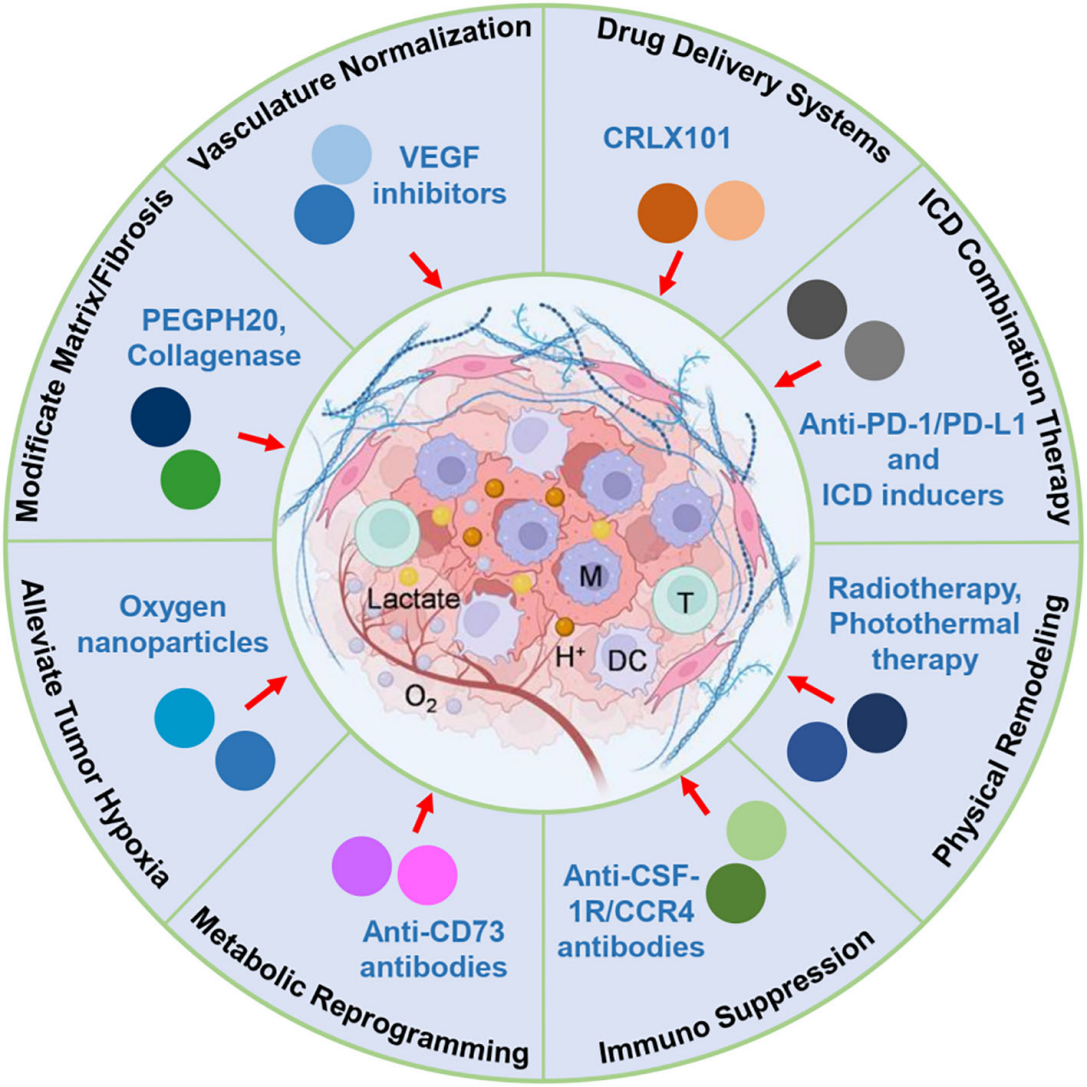
Schematic illustration of key tumor microenvironmental barriers limiting ICD efficacy and corresponding therapeutic strategies. This figure illustrates the major immunological, physical, and metabolic barriers within the solid tumor microenvironment that hinder the effectiveness of ICD-based therapies. Key immunosuppressive components include Tregs, MDSCs, and TAMs, which inhibit antigen presentation and effector T-cell responses. Physical barriers, such as dense ECM, fibrotic stroma, and abnormal vasculature, obstruct immune cell infiltration and drug penetration. Metabolic stressors, including hypoxia, glucose depletion, and accumulation of immunosuppressive metabolites (e.g., lactate, adenosine), further compromise immune function. Therapeutic strategies to overcome these obstacles are highlighted, including vascular normalization, ECM remodeling, metabolic reprogramming, immunosuppressive cell targeting, and nanomedicine-based delivery. These approaches aim to enhance immune accessibility, sustain cytotoxic activity, and improve the therapeutic outcome of ICD in solid tumors.

**Figure 1** illustrates the key tumor microenvironmental barriers—including immunosuppressive cells, dense extracellular matrix, abnormal tumor vasculature, hypoxia, and metabolic suppression—that significantly impede the effectiveness of ICD. The figure also outlines corresponding therapeutic strategies currently under clinical evaluation aimed at dismantling these barriers, thus enhancing immune infiltration, activation, and sustained antitumor immune responses. Specifically, checkpoint inhibitors and depletion agents are designed to overcome immunosuppressive cell populations such as Tregs and MDSCs; vascular normalization strategies (e.g., anti-VEGF therapy) target abnormal tumor vasculature to improve immune cell delivery; ECM-modulating agents (e.g., hyaluronidase-based therapies) reduce matrix density to facilitate T cell penetration; and metabolic modulators aim to restore nutrient and oxygen availability in hypoxic or metabolically suppressive regions of the TME. These immunological, physical, and metabolic barriers within the solid tumor microenvironment substantially limit the potential of ICD to induce effective and sustained antitumor immune responses (25). Therefore, therapeutic strategies specifically designed to address and mitigate these barriers are urgently required to enhance the clinical impact and effectiveness of ICD-based cancer immunotherapies. In the following sections, we discuss how these identified barriers inform the design of therapeutic strategies, emphasizing the direct connections between each obstacle and the corresponding interventions currently under preclinical or clinical evaluation.

## 3 Current strategies to overcome microenvironmental barriers

To effectively translate ICD into clinically successful cancer immunotherapy, numerous therapeutic strategies have been developed to specifically target and overcome the complex TME barriers. These approaches are primarily designed to reduce immunosuppression, enhance immune cell infiltration, and reverse unfavorable metabolic conditions within the solid tumor environment. To orient the reader, Sections 3.1 – 3.7 map each major barrier (vascular, stromal/ECM, hypoxic–metabolic, immunosuppressive cells, physical/ablative, and delivery constraints) to the corresponding intervention, and Section 4 synthesizes representative clinical studies that evaluate these approaches in patients.

### 3.1 Normalization of tumor vasculature

Aberrant angiogenesis creates dysfunctional tumor blood vessels, significantly limiting immune cell infiltration and therapeutic drug delivery. Therapeutic strategies, such as VEGF inhibitors (e.g., bevacizumab), have shown promise in normalizing tumor vasculature by pruning immature vessels, stabilizing vessel structures, and improving tumor perfusion (26, 27). Clinically, combining anti-VEGF therapy with immune checkpoint inhibitors enhances immune infiltration and efficacy, with early evidence from trials such as NCT02366143 (advanced renal cell carcinoma) (28, 29).

### 3.2 Modification of extracellular matrix and fibrosis

ECM deposition and tumor-associated fibrosis restrict immune cell infiltration and drug diffusion (30). Agents targeting ECM components, including collagenase and hyaluronidase enzymes, have demonstrated preclinical efficacy by enhancing immune infiltration and drug penetration (31). Notably, recombinant hyaluronidase (PEGPH20) in combination with chemotherapy or immunotherapy has entered clinical trials (NCT01839487, NCT03481920), aiming to alleviate ECM density and thus promote immune cell access and therapeutic efficacy in pancreatic and other solid tumors (32, 33). However, the phase III HALO 301 study in HA-high metastatic pancreatic ductal adenocarcinoma showed no overall survival (OS) or progression-free survival (PFS) benefit with PEGPH20 plus nab-paclitaxel/gemcitabine versus placebo plus nab-paclitaxel/gemcitabine: median OS 11.2 vs 11.5 months (HR 1.00; 95% CI 0.80 – 1.27; P = 0.97) and median PFS 7.1 vs 7.1 months (HR 0.97; 95% CI 0.75 – 1.26). Although ORR was higher (47% vs 36%), this did not translate into survival improvement, and grade ≥3 AEs (e.g., fatigue, muscle spasm, hyponatremia) occurred more frequently in the PEGPH20 arm (34).

The failure of HALO 301 to achieve survival benefit may be related to the heterogeneity of hyaluronan expression among patients, incomplete stromal remodeling, and treatment-limiting toxicities, which may have offset the potential pharmacokinetic gains from ECM targeting. This interpretation is supported by both clinical and mechanistic evidence. Subgroup analyses from earlier PEGPH20 trials demonstrated that clinical benefit was largely restricted to patients with uniformly high hyaluronan (HA) expression, whereas HALO - 301 enrolled an HA-high cohort with substantial intra- and inter-tumoral variability, potentially leading to patient misclassification and dilution of effect (32). Mechanistic studies further reveal that enzymatic depletion of HA can transiently decompress tumor vessels and enhance drug delivery, but the degree and durability of stromal remodeling in patients may be insufficient to translate into consistent survival gains (7). Additionally, higher rates of grade ≥3 adverse events in the PEGPH20 arm, including thromboembolic events, likely reduced treatment adherence and dose intensity, offsetting pharmacokinetic advantages (34).

### 3.3 Alleviation of tumor hypoxia

Hypoxia-driven metabolic adaptations profoundly suppress immune function within tumors. Therapeutics aimed at targeting hypoxia-inducible factors (HIF - 1α inhibitors) and innovative oxygen-releasing nanoparticles have been explored to mitigate hypoxia (35). Small molecules like evofosfamide (TH - 302), designed to target hypoxic regions selectively, have shown promise in preclinical and clinical studies (e.g., NCT01497444), improving immune cell functionality and tumor sensitivity to immunotherapy when combined with immune checkpoint inhibitors (36). Nevertheless, the randomized phase III SARC021 trial in soft-tissue sarcoma found that adding evofosfamide to doxorubicin did not improve OS versus doxorubicin alone; therefore, this combination cannot be recommended as first-line therapy in that setting (37). Similarly, the lack of efficacy in SARC021 may be explained by insufficient hypoxia selectivity, heterogeneous tumor oxygenation profiles, and possible limitations in drug penetration to hypoxic niches.

### 3.4 Metabolic reprogramming of the immunosuppressive microenvironment

Tumor metabolism profoundly shapes immunological responses through accumulation of immunosuppressive metabolites such as lactate, adenosine, and kynurenine. Therapeutics such as adenosine pathway inhibitors (e.g., anti-CD73, anti-A2A receptor antibodies) are actively being investigated in clinical trials (NCT02503774, NCT03367819) to neutralize adenosine-driven immune suppression (38, 39). Similarly, lactate transport inhibitors targeting monocarboxylate transporters (MCTs) have entered preclinical testing, aiming to restore immune effector cell functionality within tumors.

### 3.5 Targeting immunosuppressive cell populations

Various approaches target immunosuppressive cells such as Tregs, MDSCs, and TAMs. Anti-CSF-1R antibodies (e.g., cabiralizumab), aimed at reprogramming TAM polarization from an immunosuppressive M2 phenotype toward a pro-inflammatory M1 phenotype, are being clinically evaluated (NCT02526017, NCT02880371) (40, 41). Similarly, anti-CCR4 antibody (mogamulizumab) targeting Tregs has shown potential for enhancing immune activation in solid tumors (NCT02946671) (42).

### 3.6 Physical and ablative strategies to remodel TME

Localized physical approaches, including radiotherapy, photodynamic therapy (PDT), and photothermal therapy (PTT), have been utilized to physically disrupt the tumor architecture and induce ICD (43). Clinical evidence from radiotherapy combined with immune checkpoint inhibitors (e.g., PEMBRO-RT, NCT02492568) supports further evaluation by promoting immune cell infiltration and reversing local immune suppression through induced inflammatory responses and DAMP release (44).

### 3.7 Nanotechnology-based drug delivery systems:

Advanced nanoparticle-based delivery systems are being employed to specifically modulate the TME, enhance drug delivery, and amplify ICD efficacy. For instance, camptothecin-based nanoparticle formulations (CRLX101, NCT02769962) enhance tumor-specific ICD induction by promoting controlled intratumoral release, reducing systemic toxicity, and overcoming TME barriers through improved bioavailability and therapeutic targeting (45, 46).

These multifaceted strategies to overcome immunological, physical, and metabolic barriers demonstrate substantial potential to amplify ICD-induced antitumor immunity. Continued clinical development, optimization of combination regimens, and identification of predictive biomarkers will further improve the therapeutic efficacy of ICD in solid tumors, ultimately enhancing patient outcomes. A comprehensive overview of these therapeutic strategies, representative agents, mechanisms of action, and corresponding clinical trials is provided in **Table 1**. In the next section, we transition from mechanistic rationale to clinical translation, explicitly pairing each strategy with outcomes from representative trials.

**Table 1:** Clinical studies of stroma/ECM or TME-modulating strategies (split by outcome) with cancer type and timeline.

Type	Therapy Strategy	Cancer type	Study NCT	Year initiated	Year completed	Key outcome summary	References
Successful (positive primary endpoint or practice-changing signal)	Atezolizumab + Bevacizumab + Carboplatin/Paclitaxel (ABCP) vs BCP (IMpower150)	Metastatic non-squamous NSCLC	Phase III, NCT02366143	2015	2019	Met primary endpoints; ABCP improved OS and PFS vs BCP; led to FDA approval of atezo-bev-chemo in 1L nsq-NSCLC.	(28)
	PEGPH20 + nab-paclitaxel + gemcitabine (HALO - 202) in HA-high PDAC	Metastatic PDAC (HA-high)	Phase II, NCT01839487	2013	2018	Signal of PFS/ORR benefit in HA-high subgroup; prompted Phase III HALO - 301.	(32)
	Pembrolizumab after SBRT vs pembrolizumab alone (PEMBRO-RT)	Advanced NSCLC	Phase II, NCT02492568	2015	2018	RT→pembro arm improved out-of-field response and showed PFS/OS signals vs pembro alone.	(47)
Unsuccessful (failed primary endpoint/terminated/negative)	PEGPH20 + AG vs placebo + AG (HALO - 301)	Metastatic PDAC (HA-high)	Phase III, NCT02715804	2016	2019	No OS benefit; program halted; JCO full results published 2020.	(34)
	PEGPH20 + avelumab	PDAC (chemo-resistant)	Early-phase, NCT03481920	2018	— (terminated)	Trial terminated; no registrational signal reported.	(48)
	Cabiralizumab (CSF1R mAb) + nivolumab	Selected advanced solid tumors (incl. PDAC cohorts)	Phase 1a/1b, NCT02526017	2015	2020	Completed without practice-changing efficacy signal.	(40)
	ARRY-382 (CSF1R inhibitor) + pembrolizumab	Advanced solid tumors	Phase 1b/2, NCT02880371	2016	2019	Limited activity; development not advanced based on this study.	(41)
	T-VEC + pembrolizumab (MASTERKEY - 265) vs pembro	Unresectable/metastatic melanoma	Phase III component of NCT02263508	2014	2022	Phase 3 missed primary end point despite Phase 1b signal.	(49)
	Evofosfamide (TH - 302) + doxorubicin vs doxorubicin (SARC021)	Advanced soft-tissue sarcoma	Phase III, NCT01440088	2012	2016/2017	No OS benefit; combination not recommended.	(37)

## 4 Clinical translation and current evidence

Clinical translation of strategies targeting TME barriers to enhance ICD represents a rapidly evolving area in oncology, underscored by a growing number of clinical trials and promising therapeutic outcomes (43). Various innovative combinations designed to reshape the immunosuppressive, metabolic, and physical landscape of solid tumors have advanced into clinical evaluation, highlighting both the feasibility and therapeutic potential of TME-targeted ICD enhancement. Building on the mechanistic rationale outlined in Section 3, we connect each intervention to the specific barrier it addresses and summarize key endpoints (PFS, OS, ORR) from representative studies to clarify the strength and limitations of the evidence.

### 4.1 Combining ICD-inducing chemotherapy with microenvironmental modulators

Emerging clinical evidence suggests potential benefit from combining conventional ICD-inducing chemotherapies, such as oxaliplatin, cyclophosphamide, and paclitaxel, with TME-targeted therapies in selected populations. For example, oxaliplatin-based chemotherapy combined with immune checkpoint inhibitors and antiangiogenic agents (e.g., bevacizumab) has been investigated in metastatic colorectal cancer (NCT02375672) and pancreatic cancer (NCT04181645) (50, 51). These studies demonstrated improved immune cell infiltration, reduced immunosuppression, and enhanced overall response rates compared to chemotherapy alone, reflecting synergistic effects from simultaneous ICD induction and microenvironmental modulation. For instance, in the phase 3 IMpower150 study, atezolizumab + bevacizumab + carboplatin/paclitaxel achieved a median PFS of 8.3 vs 6.8 months and 6-month PFS rates of 71.7% vs 57.0% compared with bevacizumab + chemotherapy; subsequent analyses confirmed an overall survival advantage for the combination, albeit with effect sizes on the order of months and varying across subgroups (52). In part, the clinical success of IMpower150 may be attributed to complementary mechanisms, including VEGF inhibition–mediated vascular normalization, relief of VEGF-driven immunosuppression, and chemotherapy-induced immunogenic cell death, which together facilitate enhanced T-cell infiltration and activation.

### 4.2 Radiotherapy-induced ICD combined with immune checkpoint blockade

Radiotherapy, a potent ICD inducer, has shown preliminary or modest benefit in combination with immune checkpoint inhibitors by simultaneously alleviating physical barriers, triggering immunogenic cell death, and reducing TME-associated immune suppression. The PEMBRO-RT trial (NCT02492568) combined pembrolizumab with focal radiotherapy in metastatic non-small-cell lung cancer (NSCLC), showing increased intratumoral cytotoxic T-cell infiltration, elevated immune activation biomarkers, and improved progression-free survival (47). In PEMBRO-RT, the ORR at 12 weeks was 36% vs 18% (P = 0.07), median PFS 6.6 vs 1.9 months (HR 0.71; P = 0.19), and median OS 15.9 vs 7.6 months (HR 0.66; P = 0.16) for pembrolizumab + SBRT versus pembrolizumab alone; although numerical improvements were observed, differences did not reach statistical significance, with the largest signal seen in PD-L1-negative tumors (47). Similarly, the RADVAX study (NCT01497808) involving prostate cancer patients combined radiotherapy and ipilimumab, revealing significant immune modulation and encouraging clinical responses, thereby providing strong clinical evidence supporting this combinational approach (53).

### 4.3 Nanoparticle-based delivery systems for enhanced ICD and TME modulation

Clinical trials investigating nanoparticle formulations designed for targeted ICD induction and TME modulation represent another promising translational avenue. CRLX101, a camptothecin-based nanoparticle formulation, demonstrated enhanced ICD induction via potent DNA damage and ER stress, and has been clinically tested in combination with pembrolizumab for advanced solid tumors including ovarian and colorectal cancers (NCT02769962) (54). Preliminary results indicate improved intratumoral drug retention, enhanced immune cell recruitment, and promising safety profiles, affirming the clinical feasibility of nanoparticle-based ICD enhancement strategies. However, efficacy signals remain preliminary and no randomized phase III data are currently available for most nanoformulations; thus, any clinical benefit should be regarded as investigational pending definitive trials.

### 4.4 Oncolytic viruses and peptide-based therapies for TME remodeling

Localized ICD induction approaches such as oncolytic viruses and intratumoral peptide therapies are gaining clinical traction. Talimogene laherparepvec (T-VEC), an FDA-approved oncolytic virus, induces robust ICD through direct tumor lysis and immune activation, remodeling the local TME. Clinical trials combining T-VEC with immune checkpoint inhibitors (e.g., KEYNOTE - 034, NCT02263508) have reported substantial improvement in overall response rates and prolonged patient survival compared to historical monotherapy outcomes (55, 56). By contrast, in the randomized phase 3 MASTERKEY - 265 trial, T-VEC + pembrolizumab did not significantly improve PFS (HR 0.86; 95% CI 0.71 – 1.04; P = 0.13) or OS (HR 0.96; 95% CI 0.76 – 1.22; P = 0.74) compared with pembrolizumab plus placebo; ORR was 48.6% vs 41.3% (CR 17.9% vs 11.6%), and grade ≥3 treatment-related AEs occurred in 20.7% vs 19.5% (49). For MASTERKEY - 265, the absence of benefit despite robust intratumoral viral replication suggests that oncolytic virus–mediated immune priming alone may be insufficient in poorly immunogenic tumors, highlighting the need for improved patient selection and combination sequencing. Likewise, LTX - 315, an oncolytic peptide designed to trigger ICD and local TME modulation, demonstrated encouraging immunological responses and tumor regression in early-phase clinical studies (NCT01986426), further validating the clinical potential of localized ICD approaches (57).

### 4.5 Clinical management of toxicities and safety considerations

Although promising, these combinational strategies necessitate careful clinical management due to potential additive toxicities, particularly immune-related adverse events (irAEs). Clinical experiences from trials such as PEMBRO-RT in advanced non-small cell lung cancer (NSCLC) (NCT02492568), RADVAX in metastatic melanoma (NCT01497808), and nanoparticle-based therapies in triple-negative breast cancer (TNBC) (NCT02769962) highlight the importance of meticulous patient monitoring, precise dosing strategies, and supportive management protocols to ensure optimal safety and therapeutic effectiveness (47, 58, 59). As an example, in MASTERKEY - 265, grade ≥3 treatment-related adverse events occurred in approximately one-fifth of patients in both arms (20.7% vs 19.5%), underscoring the need for careful monitoring and patient selection when deploying TME-modulating combinations (49).

### 4.6 Summary of clinical insights

Taken together, current clinical studies suggest a potential for enhancing ICD through targeted modulation of the TME; however, the magnitude of benefit is generally modest (often measured in months), heterogeneous across tumor types and biomarkers, and not consistently accompanied by overall survival gains. Positive signals (e.g., IMpower150) coexist with neutral phase III results (e.g., HALO 301 for PEGPH20; SARC021 for evofosfamide; MASTERKEY - 265 for T-VEC + pembrolizumab), highlighting unresolved questions around patient selection, optimal sequencing/combination, and toxicity management. Consequently, biomarker-guided, randomized trials with rigorous time-to-event endpoints remain essential to define where these strategies deliver clinically meaningful benefit (25).

## 5 Challenges and concluding perspectives

Although multiple TME-targeted approaches can potentiate ICD in principle, most improvements observed clinically are incremental and become evident only over several months of follow-up. Moreover, not all mechanistically compelling strategies translate into survival benefits in randomized settings (e.g., PEGPH20 in HALO 301, evofosfamide in SARC021, and T-VEC + pembrolizumab in MASTERKEY - 265), emphasizing the need for stringent patient stratification and robust phase III validation before broad adoption (34, 49). These examples illustrate that despite strong mechanistic rationale, limitations such as inadequate biomarker-driven patient selection, treatment-related toxicities, and challenges in trial design often contribute to the lack of survival benefit, highlighting the importance of refining future clinical approaches.

Despite significant clinical progress in targeting TME barriers to enhance ICD, several critical challenges remain. Tumor heterogeneity continues to complicate therapeutic strategies, as variable patient responses demand personalized approaches guided by robust biomarkers (60). Additionally, the complexity of combinatorial regimens increases the risk of immune-related adverse events (irAEs), emphasizing the need for refined clinical management protocols (61). Translating promising preclinical findings to clinical success requires development of advanced preclinical models that accurately reflect human tumor biology and immune interactions.

Nevertheless, integrating ICD induction with precise TME modulation offers substantial promise. For clarity, this section has been revised to more clearly articulate how the preceding discussion of barriers is directly connected to the rationale for these integrated strategies. Future directions should focus on personalized therapeutic regimens based on individual tumor characteristics, innovative delivery platforms (e.g., nanoparticle technologies), and improved clinical trial designs featuring adaptive strategies and immune-specific endpoints. Interdisciplinary collaboration among clinicians, immunologists, bioengineers, and pharmaceutical scientists remains essential to addressing these challenges. Equally important, these integrated strategies—particularly the combination of ICD induction with microenvironment-targeted therapies—hold substantial clinical promise. However, their translation into routine practice will critically depend on rigorous validation in well-designed clinical trials. Ultimately, continued refinement of these integrated approaches is poised to significantly expand patient populations benefiting from ICD-based therapies, reshaping the landscape of cancer immunotherapy and substantially improving clinical outcomes.
